# Invading the dura: A complex case of high-risk cutaneous squamous cell carcinoma highlighting the crucial role of vigilant wound surveillance and timely multidisciplinary care

**DOI:** 10.1016/j.jdcr.2026.07.029

**Published:** 2026-07-24

**Authors:** Kimberly W. Lu, Samantha S. Sattler, Jordan B. Slutsky

**Affiliations:** Department of Dermatology, Stony Brook University, Stony Brook, New York

**Keywords:** atypical fibroxanthoma, BWH T3/AJCC T4b, cutaneous squamous cell carcinoma, dural invasion, Mohs micrographic surgery, non-healing surgical site, skull invasion

## Introduction

Invasion of cutaneous squamous cell carcinoma (cSCC) into skull and dura poses significant morbidity and mortality, with rates of local recurrence, nodal metastasis, and disease-specific death exceeding 80%, 50%, and 90%, respectively.^1^ We present a case of a moderately differentiated cSCC of the scalp with invasion into skull and dura (Brigham and Women’s Hospital T3/American Joint Committee on Cancer eighth edition T4b). The tumor arose within a scar of prior atypical fibroxanthoma (AFX), which had been treated with staged excision with complete circumferential peripheral and deep margin assessment (CCPDMA). Our case emphasizes the importance of close post-operative follow-up, work-up of non-healing wounds, and early coordinated multidisciplinary care in high-risk patients.

## Case

An 81-year-old male with prior non-melanoma skin cancers and field cancerization of the scalp presented with a pearly plaque on the left vertex scalp near prior Mohs scars from both a basal cell carcinoma and a squamous cell carcinoma (Fig 1, *A*). Biopsy revealed AFX. An adjacent hyperkeratotic plaque on the right vertex scalp was debrided, revealing friable, purulent granulation-like tissue. Wound culture was negative. To treat the biopsy-proven AFX, staged excision with CCPDMA was performed. The first stage resulted in a 3.8 × 2.7 cm defect to the skull with negative 0.5 cm margins confirmed by a dermatopathologist, which included a sampled portion of the right vertex scalp lesion. The defect was repaired with bovine xenograft (Fig 1, *B*).

**Fig 1:**
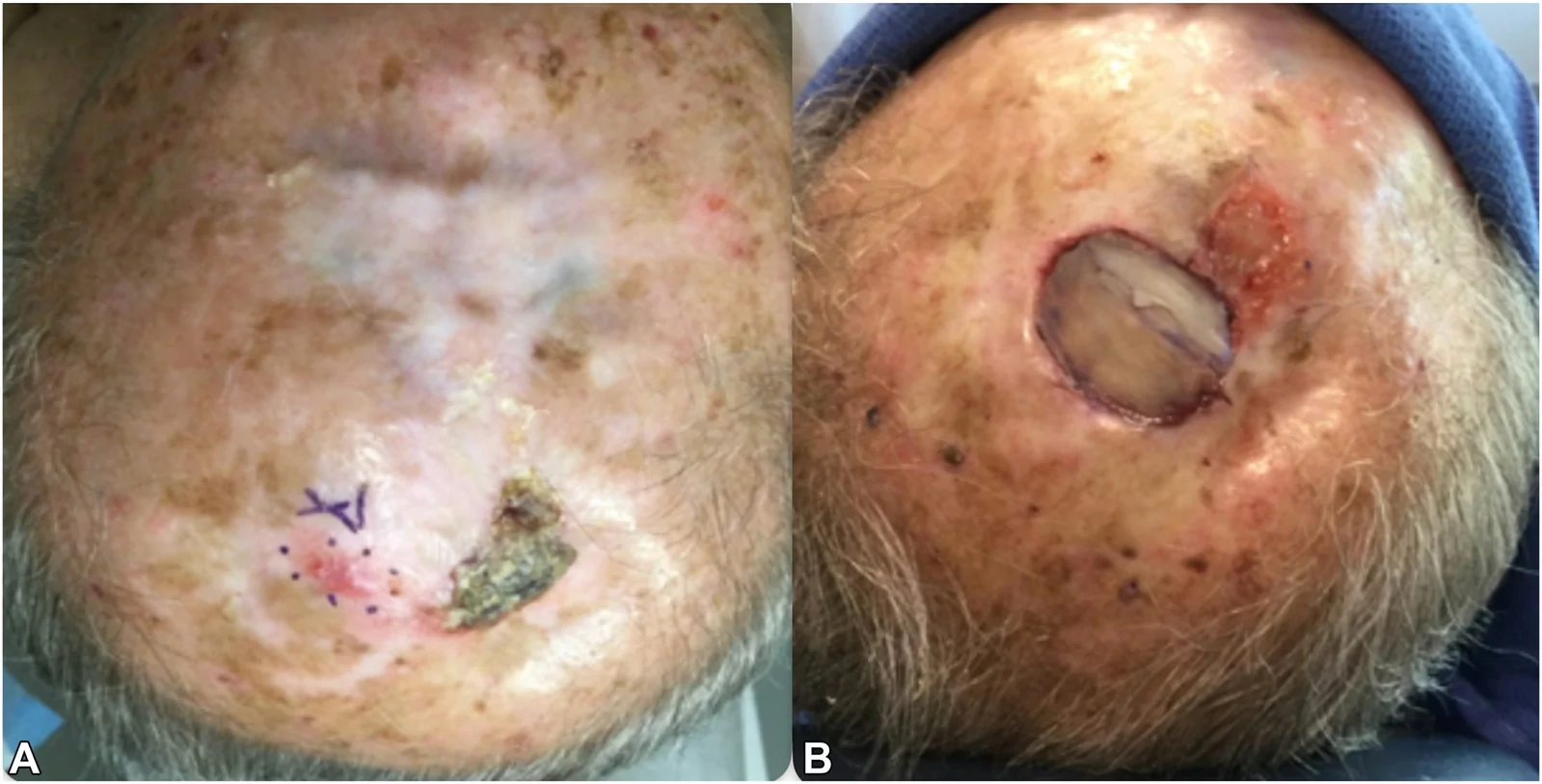
**A,** Pearly, erythematous plaque on the left vertex scalp near prior scars from a basal cell carcinoma and a squamous cell carcinoma both treated with Mohs surgery; adjacent plaque with brown and gray hyperkeratosis on the right vertex scalp. Biopsy of site A revealed atypical fibroxanthoma. **B,** Repair of scalp defect (3.8 × 2.7 cm) with bovine xenograft following resection of atypical fibroxanthoma via staged excision with CCPDMA. The adjacent lesion on the right vertex scalp was sampled during the excision and was negative for malignancy.

Five months later, the surgical wound exhibited incomplete healing and purulence. Culture grew *Pseudomonas aeruginosa* and coagulase-negative staphylococcus. After treatment with acetic acid soaks, silver sulfadiazine, and levofloxacin, wound healing resumed. Repeat culture was negative.

Given persistent granulation tissue and lack of full epithelialization 9 months post-op, the surgical site was re-biopsied, revealing fibrosing granulation tissue with no evidence of neoplasm. One-week, 2-month, and 4-month wound checks noted remnant, non-infected granulation tissue, which was treated with silver nitrate and gentian violet.

Fifteen months post-op, an ulcerated plaque was observed at the surgical site (Fig 2). Biopsy revealed moderately differentiated cSCC with CD10 failing to highlight AFX. After the first traditional Mohs stage, a 2.8 × 1.0 cm area of clinically apparent tumor invading into bone was noted within the 6.5 × 6.2 cm defect (Fig 3, *A*). Permanent sectioning confirmed infiltrative cSCC. Given bone invasion, the patient was referred to neurosurgery. CT brain revealed an 18 mm focus of outer calvarial parietal bone erosion. MRI brain did not show dural or intracranial involvement (Fig 3, *B*).

**Fig 2:**
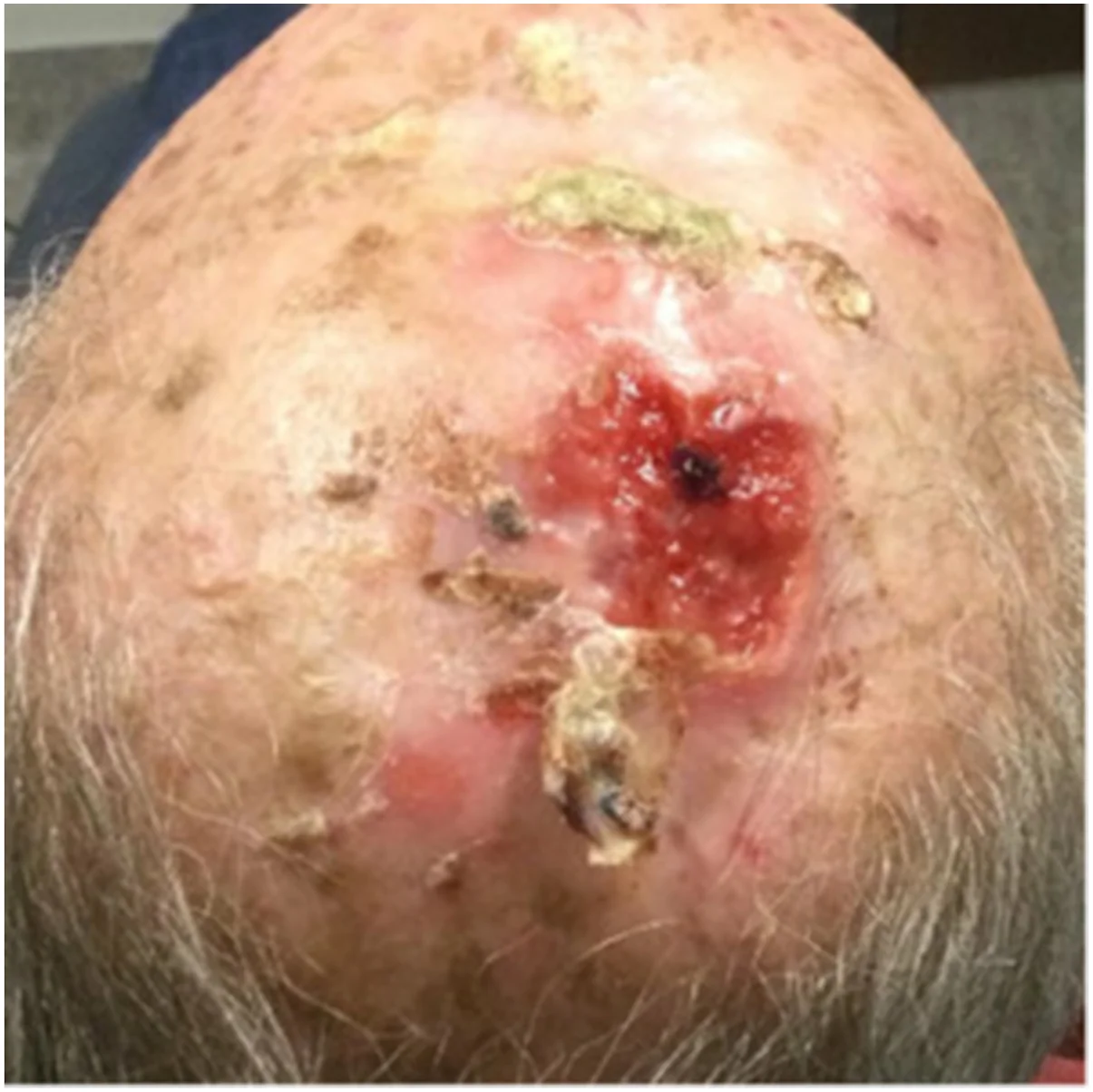
Ulcerated plaque at the site of previous atypical fibroxanthoma extirpated by staged excision with CCPDMA 15 months prior. Biopsy revealed a moderately differentiated squamous cell carcinoma with CD10 failing to highlight AFX.

**Fig 3:**
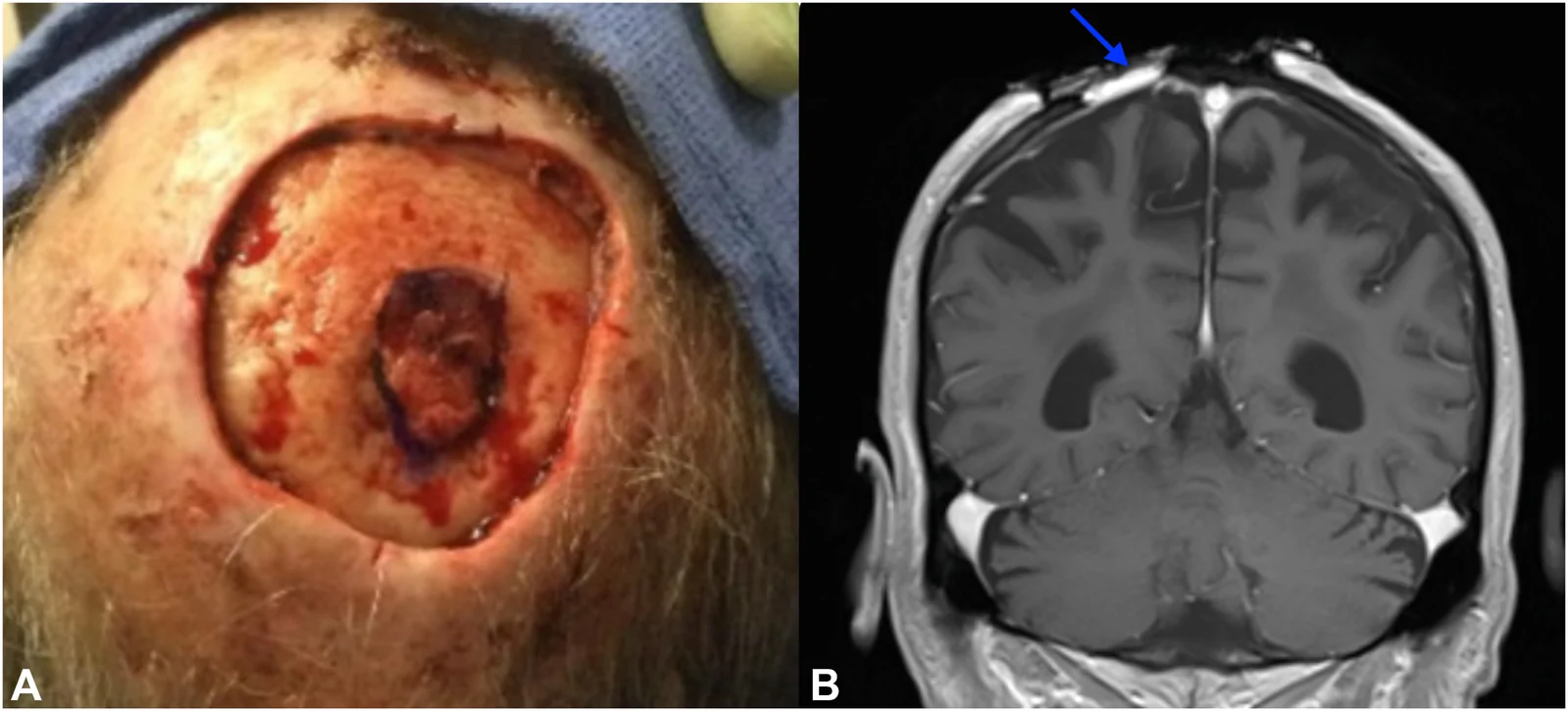
**A,** After 1 stage of traditional Mohs surgery for resection of the 4.5 × 4.9 cm squamous cell carcinoma of the scalp, a 2.8 × 1.9 cm area of clinically apparent tumor invading into bone was observed. The total Mohs defect measured 6.5 × 6.2 cm. Mohs surgery was stopped, and the patient was referred for neurosurgical intervention. **B,** MRI Brain with and without contrast demonstrated a 5 cm scalp defect of the right parietal vertex with a small 18 mm osseous defect involving the right parietal bone with intense enhancement within the osseous defect (*blue arrow*).

The patient underwent joint neurosurgery and plastic surgery. Right parietal craniotomy was performed with resection of tumor involving bone and dura. Specimens confirmed infiltrative cSCC. Some ragged-appearing involvement of the sagittal sinus was cauterized though not excised. DuraMatrix and titanium mesh cranioplasty were utilized for the bony defect repair while adjacent galea tissue transfer and a free anterolateral thigh (ALT) flap were employed for the scalp defect repair. At 4 months post-op, flap debulking was performed, followed by primary closure. Three months later, the flap was viable with good contour, hair growth, and no complications. Dermatology follow-up at 3-, 6-, and 10-months post-op revealed no cSCC recurrence.

Fourteen months post-op, new ulcerated lesions on the right temple and cheek were biopsied (Fig 4), revealing moderately differentiated SCC. CT head and neck showed subcutaneous infiltration, a new right frontal calvarial lytic lesion suspicious for metastatic disease, and a few prominent cervical lymph nodes. MRI brain did not show intracranial involvement. At multidisciplinary tumor board, the patient was deemed a poor candidate for surgical resection. He was referred to oncology/radiation oncology and initiated cemiplimab immunotherapy with possible consolidative radiation therapy (RT).

**Fig 4:**
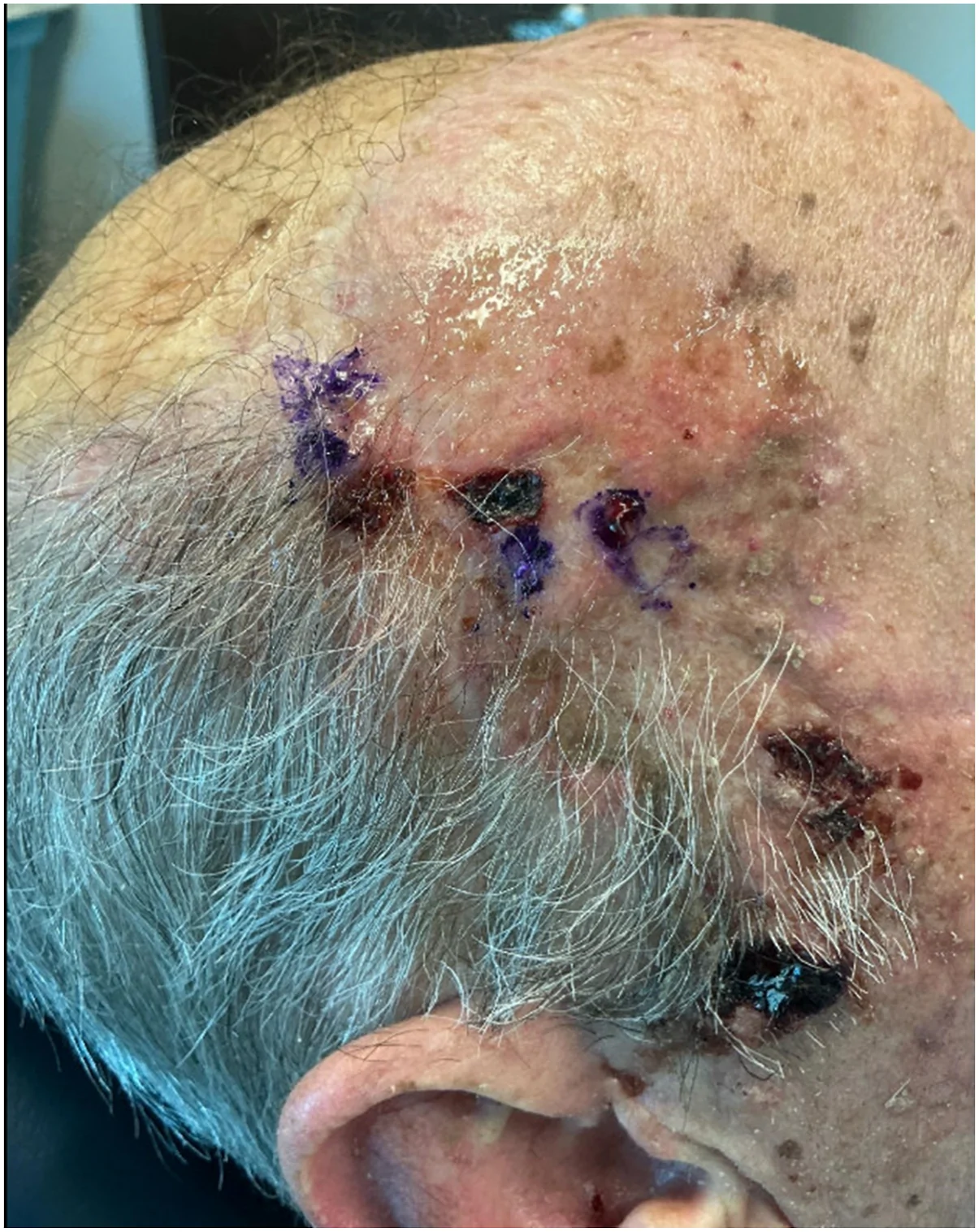
New ulcerative lesions of the right lateral cheek and right temple with dark crusted blood and serous drainage were noted at 14-months post-op, concerning for metastatic SCC. Biopsies revealed moderately differentiated SCC.

## Discussion

cSCC accounts for 16.6% of malignant scalp tumors.^1^ Risk factors for scalp cSCC include chronic scarring, advanced age, actinic damage, immunosuppression, and ionizing radiation.^2^ Common characteristics among patients with cSCC invading skull include male sex, age greater than 50, tumor location on a skull convexity, prior cutaneous scalp malignances, and moderately differentiated cSCC,^3^ which were all present in our patient.

Bone and dural invasion portend poor survival in cSCC. In a study including 680 head and neck cSCCs, skull invasion was associated with a 0% 10-year survival.^1^ In a study of 21 malignant tumors involving skull, dural invasion significantly reduced 3-year survival rates from 83% to 22%.^4^

For high-risk cSCC, the National Comprehensive Cancer Network (NCCN) recommends multidisciplinary consultation and radiologic staging with MRI and CT, followed by primary treatment with either Mohs, standard excision with wider surgical margins, or RT for non-surgical candidates.^5^ High-risk features like bone invasion may warrant adjuvant RT, followed by possible cemiplimab immunotherapy.^5^

In this case, 2 factors contributed to disease complications. First, diagnosis of cSCC was delayed despite a negative surgical stage extending into the tumor as well as a negative initial biopsy, highlighting the need for a low threshold to re-biopsy non-healing wounds. Second, delayed initiation of adjuvant RT and immunotherapy, due to concerns regarding healing after complex resection and flap reconstruction, likely facilitated recurrence with osseous metastases. This delay reflects the lack of cSCC-specific data on the optimal timing of adjuvant external beam RT (EBRT).

Although the timing of adjuvant EBRT is not well-defined in cSCC-specific guidelines, adjuvant brachytherapy, if appropriate, is recommended within 4-8 weeks of resection.^5^ NCCN head and neck cancer guidelines advise initiating adjuvant EBRT within 6 weeks regardless of reconstruction.^6^ Delays beyond 6 weeks are associated with worse outcomes,^7^^,^^8^ yet remain common. In 1 head and neck cohort, only 43% of patients received adjuvant EBRT within the recommended window; flap dehiscence accounted for delays in just 5.7% of cases, with most delays attributable to health system or patient factors.^9^ No patients experienced surgical complications during RT. Thus, although RT can accelerate flap atrophy and fibrosis, modern flap delineation techniques help mitigate toxicity.^10^ These data support early initiation of adjuvant RT for cSCC with bone or dural invasion.

This case highlights the potential for recurrent or concurrent malignancies in high-risk patients, the importance of frequent surveillance and persistent work-up of non-healing wounds, and the necessity of early multidisciplinary collaboration to prevent treatment delays.

## Conflicts of interest

None disclosed.
